# Co-production of 1,3-propanediol and phage phiKpS2 from the glycerol fermentation by *Klebsiella pneumoniae*

**DOI:** 10.1186/s40643-024-00760-w

**Published:** 2024-05-09

**Authors:** Suyang Duan, Zhirong Zhang, Xiaoli Wang, Yaqin Sun, Yuesheng Dong, Lina Ren, Lili Geng, Zhilong Xiu

**Affiliations:** 1 School of Bioengineering, Dalian University of Technology, Linggong Road 2, Dalian, 116024 P. R. China; 2 Department of Respiratory, Affiliated Dalian Municipal Central Hospital of Dalian University of Technology, Dalian, 116033 Liaoning P. R. China

**Keywords:** *Klebsiella pneumonia*, 1,3-Propanediol, Bacteriophage, Co-production, Salting-out extraction

## Abstract

As an alternative to antibiotics in response to antimicrobial-resistant infections, bacteriophages (phages) are garnering renewed interest in recent years. However, the massive preparation of phage is restricted using traditional pathogens as host cells, which incurs additional costs and contamination. In this study, an opportunistic pathogen, *Klebsiella pneumoniae* used to convert glycerol to 1,3-propanediol (1,3-PDO), was reused to prepare phage after fermentation. The phage infection showed that the fed-batch fermentation broth containing 71.6 g/L 1,3-PDO can be directly used for preparation of phage with a titer of 1 × 10^8^ pfu/mL. Then, the two-step salting-out extraction was adopted to remove most impurities, e.g. acetic acid (93.5%), ethanol (91.5%) and cells (99.4%) at the first step, and obtain 1,3-PDO (56.6%) in the top phase as well as phage (97.4%) in the middle phase at the second step. This integrated process provides a cheap and environment-friendly manner for coproduction of 1,3-PDO and phage.

**Graphical abstract:**

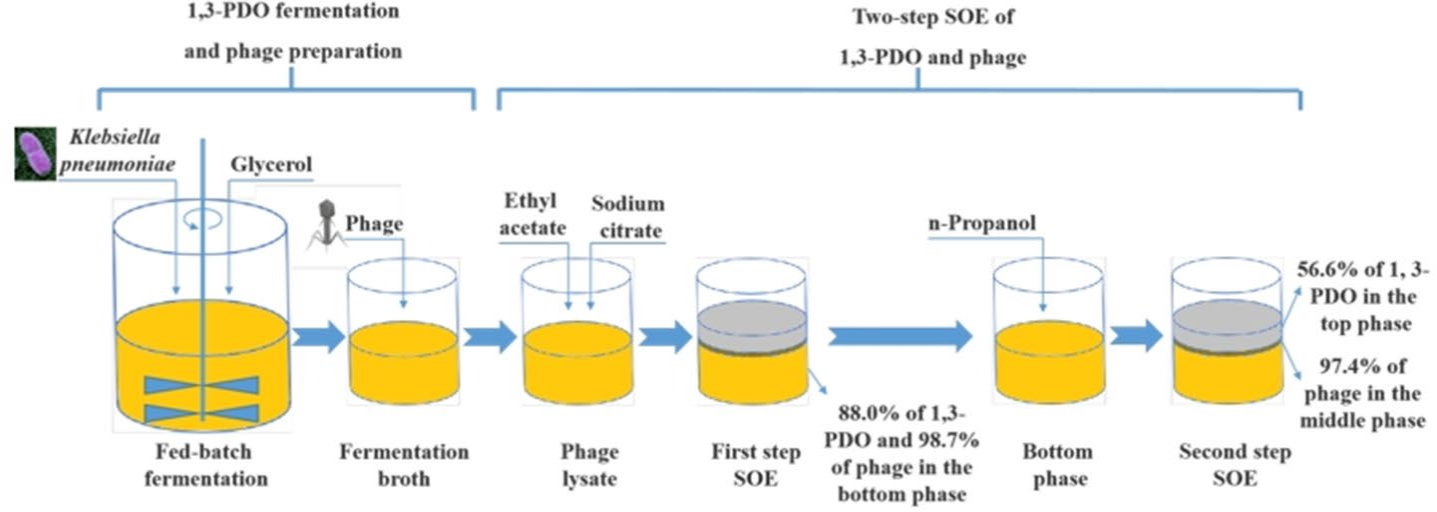

**Supplementary Information:**

The online version contains supplementary material available at 10.1186/s40643-024-00760-w.

## Introduction

At present, antimicrobial-resistantance caused by uncontrolled application of antibiotics has posed one of the serious international threats to public health and microecological balance (Hassan et al. 2021). *Klebsiella pneumoniae*, particularly Carbapenem-resistant *K. pneumoniae* (CRKP) (Effah et al. 2020), is one of clinically significant Gram-negative bacteria that have acquired much public concern, due to its potential risk in inducing broad hospital-acquired disease associated with septicemia, pneumonia, and urinary tract infections (Serna-Galvis et al. 2020). Development of effective drugs against bacterial infections is a great challenge of the twenty-first century due to the long and complicated process. In the fight against bacterial resistance, phage is a specific, natural and safe candidate (Zurabov and Zhilenkov 2021). However, the laboratorial preparation for most phages is difficult to meet the continuously increasing demand for phage therapy. Therefore, new approaches for massive phage preparation are required, by which phage stocks can be quickly and efficiently produced with high titer and high purity.

As the first step for phage preparation, host/pathogenic cell cultivation is usually carried out under specific culture conditions (Zhang et al. 2020), which not only takes a long time, but also is very costly. Some opportunistic pathogens, e.g. *Escherichia coli* and *K. pneumoniae*, have been used in the industrial production. Our team recently reported that a recombinant *κ*-Carrageenase in *E. coli* C2 could be released by phage T7, and the co-production of both products was achieved by a salting-out extraction system (Chen et al. 2023). 1,3-PDO is an important chemical intermediate for the synthesis of many polymers, which has a wide application in food, cosmetics, pharmaceutical and textile industries (Lama et al. 2020). It has been produced by *K. pneumoniae* on the capacity of 10,000 to 20,000 tons/year in three plants of China, e.g. Shenghong Group Holdings Ltd. After fermentation, both bacteria are treated as waste by burning or complete hydrolysis due to their opportunistic pathogenicity (Xiao and Ju 2018). In fact, they are the potential hosts for phage preparation. However, there is no study on the preparation of phage using waste bacteria. Therefore, we suppose it is feasible that waste bacterial cells were utilized as host cells to prepare phages after microbial production of 1,3-PDO from glycerol using *K. pneumoniae*, which would undoubtedly provide an economic co-production of two bio-products, i.e. 1,3-PDO and phage, on a large scale.

An ideal integrated bioprocess for co-production of 1,3-PDO and phage should not only meet the requirements of phage preparation using the waste *K. pneumoniae* cells after fermentation, but also separate 1,3-PDO effectively from phage in crude phage lysate. The conventional separation of 1,3-PDO from fermentation broths, including steam evaporation (Ames 2002), vacuum distillation (Zhang et al. 2021), ion-exchange chromatography (Zheng et al. 2020) and reactive extraction (Li et al. 2009b), is difficult to adapt to simultaneous separation of phage. On the other hand, downstream processing of phages is a complex, time-consuming and expensive process, including precipitation (Salim et al. 2021), centrifugation (Jacinto et al. 2018), filtration (Salim et al. 2021), chromatographic techniques (Clavijo et al. 2019), and so on. An alternative operation is a multi-step combination with above several methods, but it would complicate the purification process and increase costs (Jacinto et al. 2021). Therefore, it is urgent to develop a novel integrated process for the simultaneous separation of 1,3-PDO and phage.

Extraction is a separation unit used mostly for integrated designs. Salting-out extraction (SOE), also sometimes called as aqueous two-phase extraction (ATPE), can achieve the selective separation of target molecules (Li et al. 2019b), which is based on an aqueous two-phase system (ATPS) composed of hydrophilic solvents and salts. A few traditional ATPSs, such as ionic liquid (IL)-salt and polyethylene glycol (PEG)-salt ATPS, were used to separate *E. coli* phage T4 (Negrete et al. 2007) or M13 (Jacinto et al. 2018) and *Salmonella* phage ϕSan23 (Clavijo et al. 2019). However, these ATPSs constituted of PEG or ILs were unfavorable for the subsequent process due to high viscosity or toxicity (Oppermann et al. 2011). Our team tried to separate and purify *Klebsiella* phage by using two-step SOE systems of ethyl acetate, n-propanol and salt (Zhang et al. 2020). According to our experience, two-step SOE might be available to achieve simultaneous separation of 1,3-PDO and phage.

The objective of this study would be to set up an integrated bioprocess, in which phage was prepared by co-cultivating the fermentation broth or waste bacterial cells with phage suspension after fermentation of 1,3-PDO by *K. pneumoniae*, and two-step SOE was employed to separate the 1,3-PDO and phage simultaneously.

## Materials and methods

### Materials

1,3-PDO standard was purchased from Shanghai Aladdin Bio-Chem Technology Co., Ltd (Shanghai, China). Bicinchoninic acid (BCA) protein assay kit was provided by Shanghai Sangon Biotech Ltd (Shanghai, China). End-Point Chromogenic Endotoxin Test LAL Kit (EC80545) was bought from Xiamen Bioendo Technology Co., Ltd. (Xiamen, China). All other chemicals, such as trisodium citrate dehydrate and ethyl acetate were of analytical grade and bought from Sinopharm Chemical Reagent Co., Ltd (Shanghai, China). Seed and fermentation media had been described in the previously reported work (Shen et al. 2016). *K. pneumoniae* S2 and the phage phiKpS2 used in this work were isolated from 1,3-PDO fermentation broth (Liu et al. 2007; Shen et al. 2016). *K. pneumoniae* strain was maintained, and they were maintained at -80 °C and 4 °C, respectively.

### Fed-batch fermentation

Two fed-batch fermentations with 10% inoculum were performed in a 5 L bioreactor containing 2 L of fermentation medium at the glycerol concentration of 40 g/L. To create an anaerobic environment, nitrogen was sparged for 1 h before inoculation. The whole fermentation was carried out at 37 °C, 250 rpm, and pH 7.0 adjusted with 5 mol/L NaOH solution, as well as the residual glycerol concentration in fermenter was maintained at 15–20 g/L by adding glycerol with a purity of 96% according to the real-time consumption of glycerol (Fig. S1).

The first fed-batch fermentation lasted for 18 h, and the host bacterial pellet precipitates at different time points were collected by centrifugation for the selection of phage infection medium. When the glycerol was no longer consumed, namely the fermentation progressed to 35 h, the second fed-batch fermentation was completed. The fermentation samples at different time intervals were collected for other analytical tests.

### Phage preparation

Phage preparation was carried out by the phiKpS2 infection of *K. pneumoniae* S2 in suitable medium at an optimal multiplicity of infection (MOI). MOI was optimized as previously described method (Shen et al. 2012) in order to obtain the highest titer for phages and decrease the bacterial concentration. Briefly, the culture sample at the end of the second fed-batch fermentation (35 h) was centrifuged at 10,000 rpm for 10 min to collect the bacterial pellet precipitates. Under 37 °C, *K. pneumoniae* cells were then co-cultured for 2.5 h with phage phiKpS2 at different MOIs (0.002, 0.01, 0.05 and 0.1) in 50 mL of fresh culture medium containing glycerol of 40 g/L. The culture broth, namely the crude phage lysate containing phage, released proteins, endotoxins and other impurity, was collected to measure phage titers.

The suitable medium for phage infection was determined according to phage titer in the crude phage lysate after 2.5 h of phage infection at 37 °C and an optimal MOI. Bacterial pellet precipitates were obtained from the culture sample at the end of the first fed-batch fermentation. The corresponding infection media were fermentation medium with a glycerol concentration of 40 g/L and seed medium with 5 g/L or 20 g/L of glycerol.

To achieve co-production of 1,3-PDO and phages, the fermentation broths during the second fed-batch fermentation were collected to evaluate the availability of *K. pneumoniae* cells for phage infection in the suitable medium or direct incubation. Direct incubation was conducted by adding phiKpS2 directly into the fermentation broth and incubating at 37 °C for 2.5 h without centrifugation for bacterial precipitate and adding new medium.

### Two-step SOE of phage and fermentative products

Two-step SOE was performed according to the previously reported work (Zhang et al. 2020). Firstly, 1.0 g of sodium citrate was added into the 6.0 g of crude phage lysate. The mixture was vortexed gently for two minutes until the salt dissolved thoroughly, and the pH was adjusted to 7.2 using hydrochloric acid and sodium hydroxide solutions. Subsequently, 3.0 g of ethyl acetate was added into the mixture to form three-phase extractions. After 10 min of centrifugation at 2000 rpm, the top and bottom phases were collected, and the aggregate between two phases was suspended evenly in 1.0 mL of sterilized water. The volumes of top and bottom phase were determined by a graduated test tube, and the volume of middle phase was approximately determined by a 0.5 mL graduated centrifuge tube. Based on our previous study, a second step SOE system was built by adding 31% n-propanol (2.5 mL) to the first step bottom phase (5.5 mL). (Zhang et al. 2020). According to the procedure of the first step SOE, 1.0 ml of three phases were respectively collected for the following determination.

Partition coefficient (*K*) of 1,3-PDO and phase ratio (*Φ)* are calculated using the following equation:

1 $$K=\frac{C}{C{\prime }}$$

2 $$\varPhi =\frac{V}{V{\prime }}$$

where *C* and *C’* are the equilibrium concentrations of 1,3-PDO in 1,3-PDO-rich phase and 1,3-PDO-poor phase, respectively. *V* and *V’* are the equilibrium volumes of 1,3-PDO-rich phase and 1,3-PDO-poor phase, respectively. The 1,3-PDO-rich and 1,3-PDO-poor phase are the bottom and top phase in the first SOE step, and the 1,3-PDO-rich and 1,3-PDO-poor phase are the top and bottom phase in the second SOE step, respectively.

In the first step SOE, the recovery (*Y*_I_) of phage and 1,3-PDO is separately defined as Eq. (3). In the second step SOE, the recovery (*Y*_II_) of 1,3-PDO and phage are separately assessed with Eqs. (4) and (5). The final recovery (*Y*_f_) of 1,3-PDO and phage are evaluated by Eq. (6).

3 $${Y}_{i,I}=\frac{{C}_{bI}{V}_{bI}}{{C}_{0}{V}_{0}}$$

4 $${Y}_{II,\text{1,3}-PDO}=\frac{{C}_{tII}{V}_{tII}}{{C}_{bI}{V}_{bI}}$$

5 $${Y}_{II}phage=\frac{{C}_{mII}{V}_{mII}}{{C}_{bI}{V}_{bI}}$$

6 $${Y}_{f}={Y}_{I}{Y}_{II}$$

where *C*_t_, *C*_b_ and *C*_m_ are the measured concentration of 1,3-PDO or phage titer in the top, bottom and middle phase, respectively. *V*_t_, *V*_b_ and *V*_m_ are the volumes of top, bottom and middle phases, respectively. I and II in the equation are used to distinguish between the first and second step SOE.

During the two-step SOE, the removal rates of impurities in phage lysate are calculated based on Eqs. (7) and (8). Other products removal rates in the fermentation broth are estimated by Eqs. (7) and (9). The final removal rates (*R*_f_) of the total proteins, cells, endotoxins and other fermentation products are evaluated using Eq. (10).

7 $${R}_{i,I}=1-\frac{{C}_{i,bI}{V}_{i,bI}}{{C}_{0}{V}_{0}}$$

8 $${R}_{i,II}=1-\frac{{C}_{i,mII}{V}_{i,mII}}{{C}_{i,bI}{V}_{i,bI}}$$

9 $${R}_{i,II}=1-\frac{{C}_{i,tII}{V}_{i,tII}}{{C}_{i,bI}{V}_{i,bI}}$$

10 $${R}_{f}={R}_{i,I}+{R}_{i,II}-{R}_{i,I}{R}_{i,II}$$

where ‘i’ represents total proteins, cells and endotoxins and other fermentation products.

Concentration factor (CF) in the first step SOE is determined through dividing the volume of original crude phage lysate by the bottom phase volume (*V*_bI_). The *V*_bI_ is then used to divide by the volume of middle phase in the second step SOE to calculate the CF in the second step SOE. Total CF is equal to the product of two CFs in two-step SOE. The purification factor (PF) was determined with reference to previous work (Zhang et al. 2020).

### Analytical methods for determination of important parameters

HPLC analysis (Waters 600E) of fermentation products, including 1,3-PDO, lactic acid, glycerol, formic acid, acetic acid, 2,3-butanediol and ethanol, was performed on an Aminex HPX-87 H column (300 × 78 mm, Bio-Rad) coupled to a differential refractometer (RI Waters 410). The mobile phase is a dilute H_2_SO_4_ solution of 5 mmol/L at a flow rate of 0.6 mL/min. The injection volume of sample is 5.0 µL, and the temperature is controlled at 65 °C for column and 35 °C for detector.

Phage titer was determined according to spot test (Clavijo et al. 2019; Zhang et al. 2020).

The biomass (OD_650_) of cells in three phases of SOE was evaluated by a UV/VIS Spectrophotometer (Jasco, V-560, Japan).

Protein concentration in top, middle and bottom phases of SOE was estimated by BCA assay. Briefly, 5 µL of each sample was added to 200 µL of BCA working reagent in a 96-well microplate, and then incubated at 37 °C for 30 min. Eventually, OD_562_ was measured on a microplate reader (SpectraMax® M2e, USA). Bovine Serum Albumin (BSA) was used as standard protein at a concentration range from 25 to 1000 µg/mL.

End-point Chromogenic Limulus Amebocyte Lysate Test was used to detect the endotoxin according to the previously reported work (Zhang et al. 2020).

### Statistical analysis

All data were represented in mean ± standard error of mean (*n* = 3). Statistical analysis was accomplished by SPSS (version 18.0, Chicago, USA). Two-way ANOVA was used to evaluate the impacts of different media and different time of the first fed-batch fermentation on phage titer.

## Results and discussion

### Determination of MOI

**Fig. 1:**
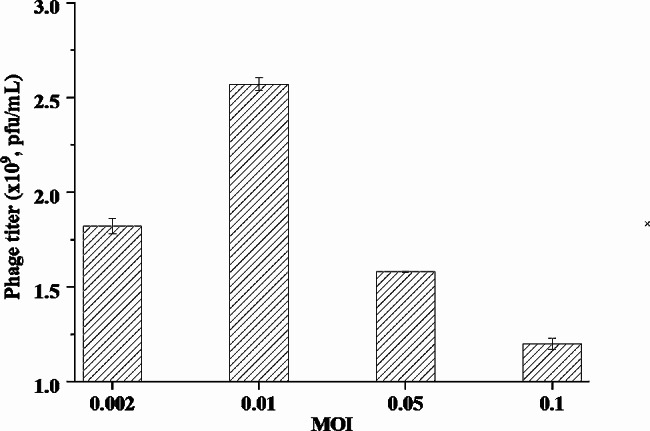
Determination of multiplicity of infection (MOI) of phages to the cell precipitate at the end of the second fed-batch fermentation (35 h)

The efficiency of phage propagation was dependent on an appropriate ratio of phage to bacterial cells, the so-called MOI (Lau et al. 2012). In this research, the capacity of phiKpS2 to infect host cells at the end of the second fed-batch fermentation (35 h) with different MOIs was assessed as shown in Fig. 1. As the MOI increased from 0.002 to 0.1, the phage titer exhibited a tendency to increase first and then decrease. The maximum titer (2.57 × 10^9^ pfu/mL) was obtained at MOI of 0.01, indicating that more progeny phages were produced than other cases. Therefore, the optimal MOI of phiKpS2 for *K. pneumoniae S2* infection was considered to be 0.01. Although a higher dose of phage was better for a faster decline in the number of host cells, it is not absolutely necessary for lysis (Shen et al. 2012), as the lower phage titer detected in this study, i.e. 1.58 × 10^9^ and 1.2 × 10^9^ pfu/mL at MOI of 0.05 and 0.1, respectively. Similar observations were also reported on several phages, such as *Acinetobacter baumannii* phage Økm18p (Shen et al. 2012), *E. coli* O78 : K80 phage ØEC1 (Lau et al. 2012) and *S. enteritidis* phage LSE7621 (Liu et al. 2020). This may be attributed to the lack of new phage progenies for continuous infection at higher MOIs, and self-replication of the phage at lower efficiency (Lau et al. 2012). According to the reports of Rabinovitch (Rabinovitch et al. 2003) and Abedon (Abedon 2011), this phenomenon occurred during infection with high MOI, where the bacterial cells are attacked by a large number of phages, leads to premature lysis of the host cells without the release of free phage progeny.

### Condition optimization of phage infection to bacterial pellet precipitates

**Fig. 2:**
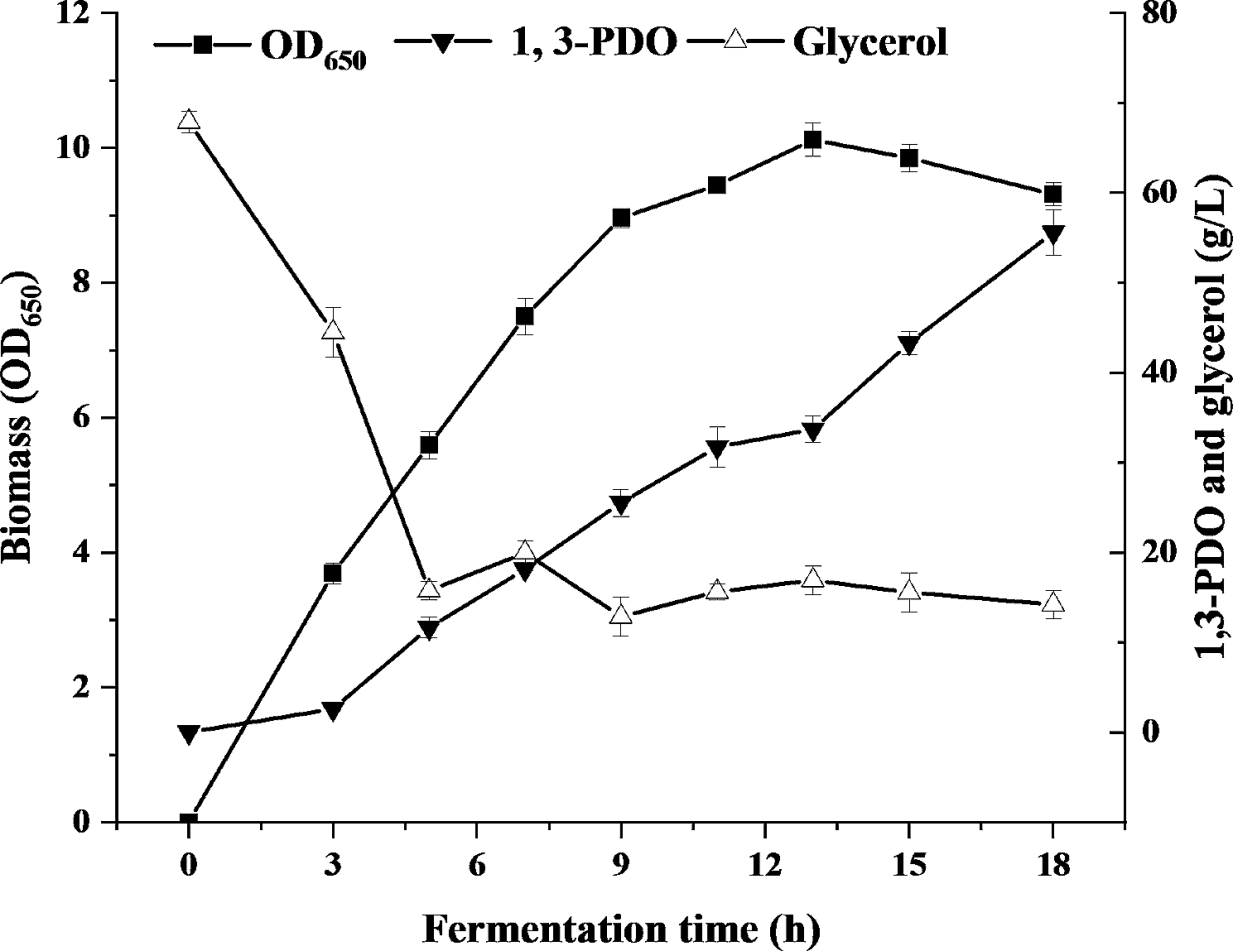
The time course of biomass (OD_650_), 1,3-propanediol (1,3-PDO) and glycerol concentration during the first fed-batch fermentation

**Fig. 3:**
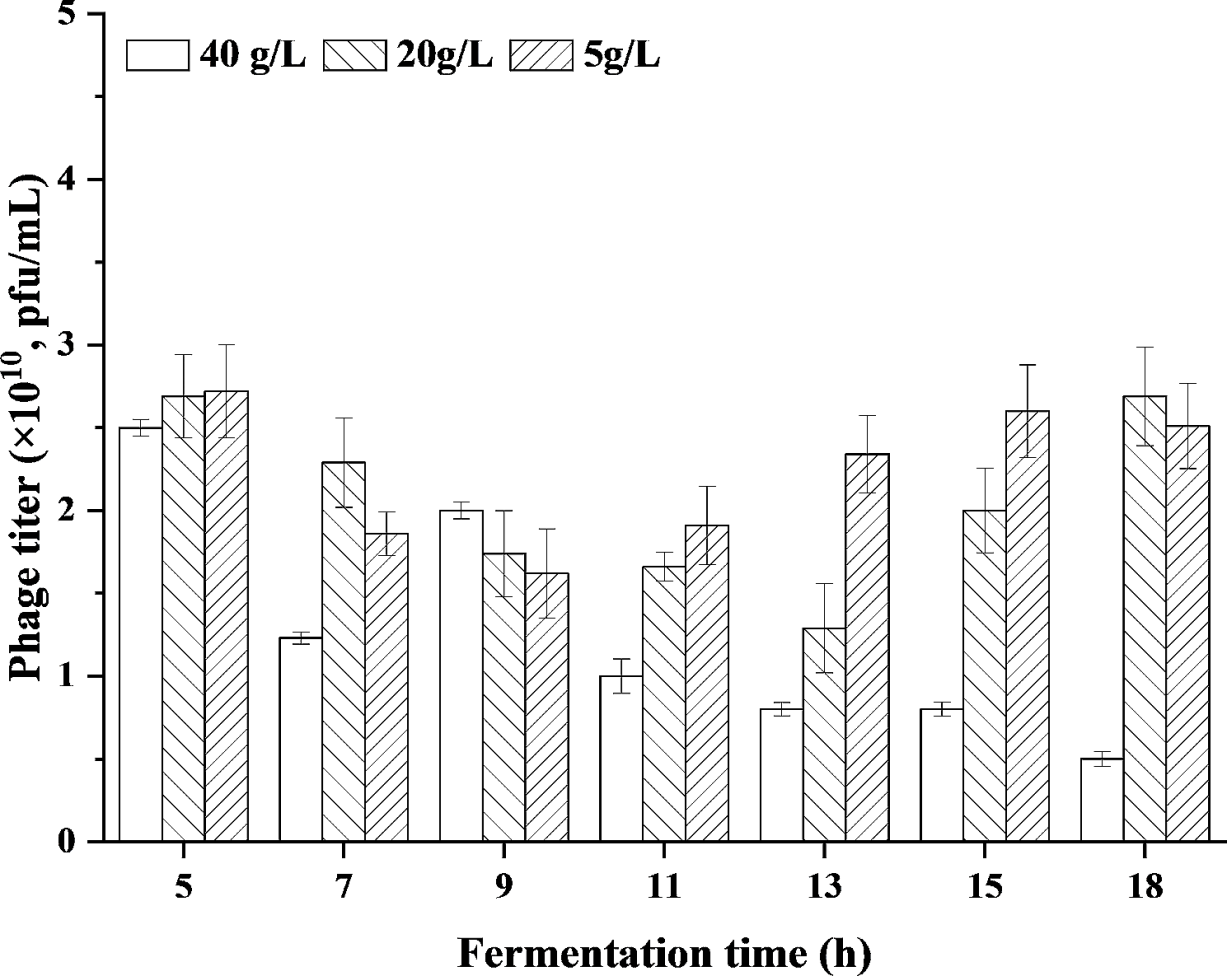
Effects of different media on phage titer obtained by phages infection to cell precipitates obtained at different time points of the first fed-batch fermentation. The different media were fermentation medium containing 40 g/L glycerol and seed medium with 20–5 g/L glycerol, respectively

As viruses, phages require the involvement of live bacteria with certain metabolic activity to propagate (Gill and Hyman 2010). Generally, phages are allowed to replicate in the host cells when the host cells and phages are inoculated together in a suitable nutrient medium for the growth of the host cells. In other words, the medium composition and the substrate limitation will affect the physiology of bacterial cells and cause the halt of phage formulation (Jończyk-Matysiak et al. 2019). LB medium containing tryptone (10 g/L), yeast extract (5 g/L), and NaCl (10 g/L) is mostly used to prepare phages in reported works, which is not suitable for phage preparation on a large scale due to high price of tryptone and yeast extract. So cheap fermentation and seed media for 1,3-PDO production were investigated for phage preparation in this study. The infectivity of phiKpS2 incubated in the fermentation medium containing 40 g/L glycerol and seed medium with 5–20 g/L glycerol were compared in this study, which were usual concentrations for glycerol fermentation and seed cultivation (Chilakamarry et al. 2022; Unrean et al. 2012; Zhang et al. 2007). Bacterial pellet precipitates used in this process were obtained from the first fed-batch fermentation, during which the biomass (OD_650_) in the fermentation broth remained at a high level, and the 1,3-PDO concentration was relatively low (Fig. 2). As shown in Fig. 3, the phage titer prepared in the seed medium was significantly higher than that in the fermentation medium in most cases (Table S1), except for seed medium with 20 g/L glycerol (5, 9 and 13 h) and seed medium with 5 g/L glycerol (5 and 9 h). When the fermentation time was extended from 5 h to 18, the phage titer obtained in fermentation medium showed a trend of decreasing (5–7 h), then increasing (7–9 h) and then decreasing (9–18 h), which may be associated with the physiological status of host cells. A decrease in phage titer was due to that the host cells grown in the early phase was prone to phage infection because of the weak cell wall, while as the fermentation entered the late phase, the bacterial cells were in an senile state and DNA started to partly degrade, affecting the production of phage (Dong et al. 2013). The phage titer began to rise slowly, which may be because the bacteria started to focus on the production of fermentation products and suppress this emergence of phage resistance. In biology, “Genetic trade-off” is a common phenomenon. When a certain trait of a bacterium changes, its adaptability will correspondingly change, which will affect the performance of other traits (Chan et al. 2016). However, the phage titer obtained in the two seed media did not change significantly in most cases (Table S2), except for seed medium with 20 g/L glycerol (13 h vs. 5 h, 13 h vs.18 h) and seed medium with 5 g/L glycerol (5 h vs.9 h) (Fig. 3). For example, at final stage of the first fed-batch fermentation (18 h) (Fig. 2), the phage titer obtained in fermentation medium containing 40 g/L glycerol was 0.5 × 10^10^ pfu/mL, while 2.69 × 10^10^ pfu/mL in seed medium containing 20 g/L glycerol and 2.51 × 10^10^ pfu/mL in seed medium containing 5 g/L glycerol (Fig. 3). This illuminated that seed medium was not only more conducive to the growth of host cells relative to fermentation medium, but also possessed a positive effect on the proliferation of phage, which may be due to that seed medium with low concentration of substrate has less osmotic shock to cells. In addition, it was noticed that the phage titer obtained in seed medium containing 5 g/L or 20 g/L glycerol was basically same in most cases (Table S1), except for 13 h. So both of them can be used for phage infection (Fig. 3). In order to reduce the waste of resources and simplify the following downstream processing, thereby control the production costs, seed medium with 5 g/L glycerol was chosen as the medium for phage infection.

### Co-production of 1,3-PDO and phage by fed-batch fermentation

**Fig. 4:**
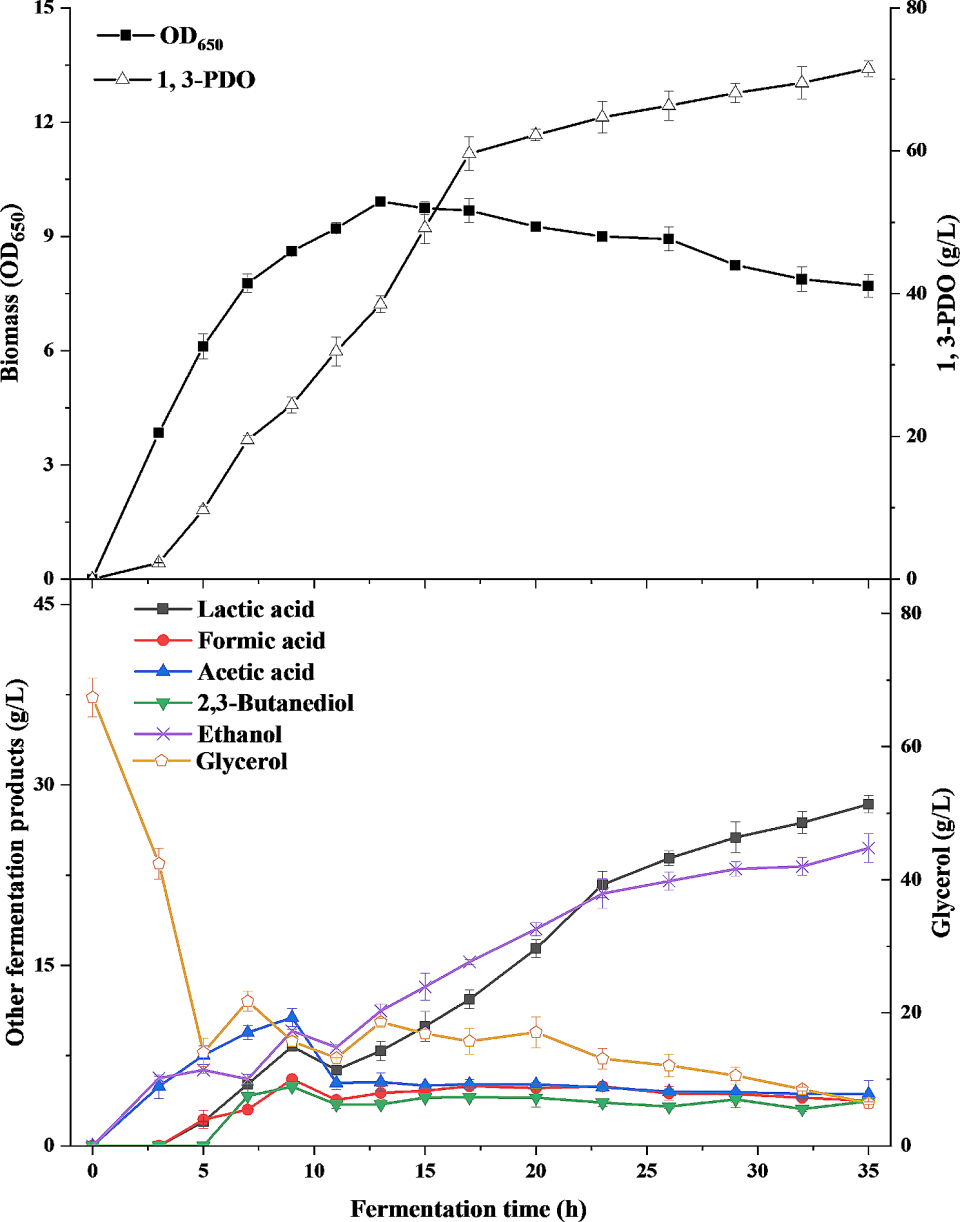
The time course of biomass (OD_650_), concentrations of glycerol and fermentative products during the second fed-batch fermentation

The second fed-batch fermentation results in higher final 1,3-PDO concentration and higher productivity, but the biomass has declined. Changes in biomass, concentrations of substrate and products during this fermentation periods (0 ∼ 35 h) were recorded in detail (Fig. 4). The *K. pneumoniae* cells grew to a maximum biomass (OD_650_ of 9.92) at 13 h, and 1,3-PDO was produced at a final concentration of 71.6 g/L and a productivity of 2.04 g/L/h at the end of the second fed-batch fermentation (35 h).

**Fig. 5:**
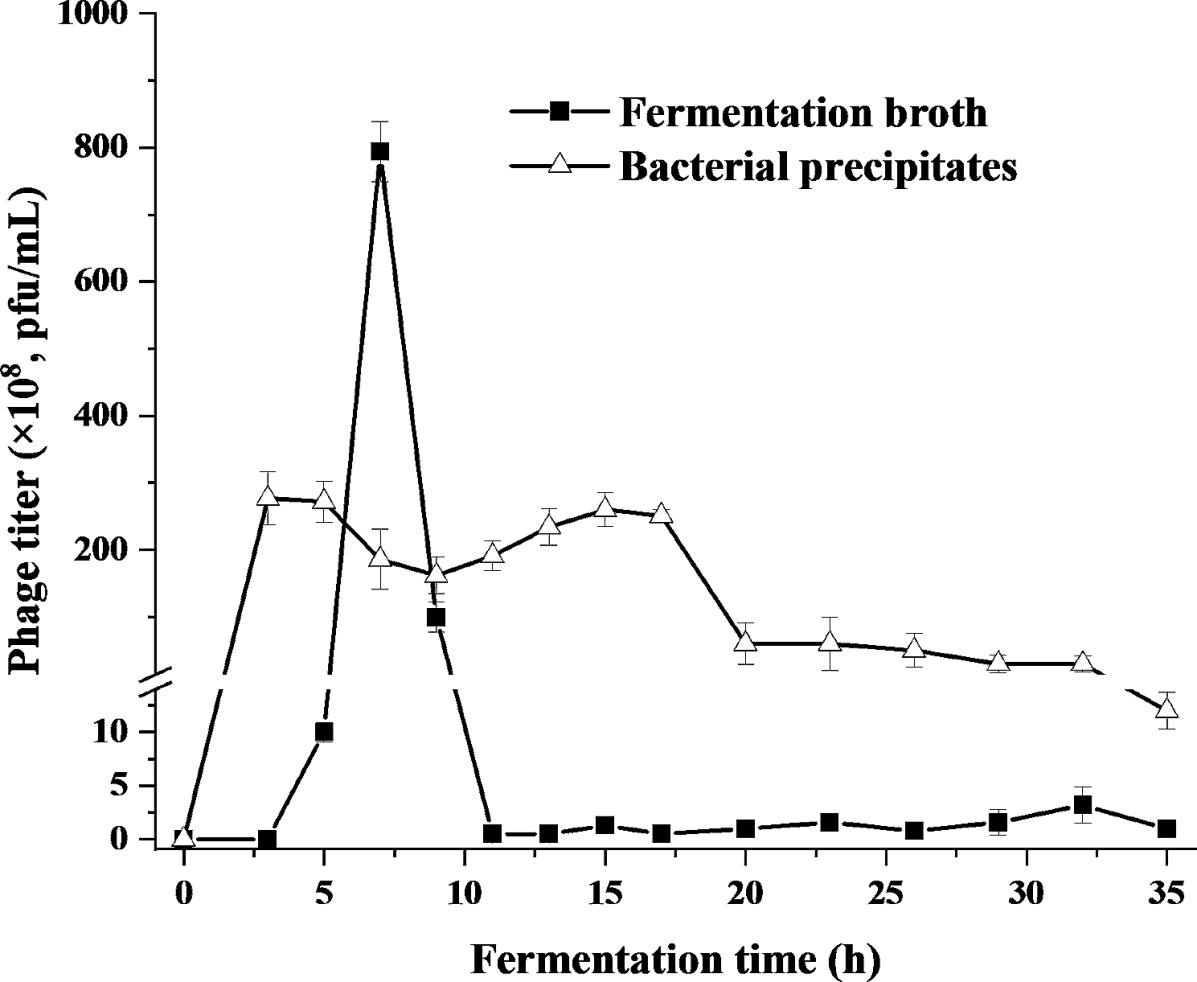
The phage titer obtained by phage infection to bacterial precipitates or fermentation broth at different time points of the second fed-batch fermentation

Bacterial pellet precipitates gathered at different fermentation time were used for phage infection in seed medium with 5 g/L glycerol as displayed in Fig. 5. Although the MOI was consistent, the phage titer still changed significantly, which may be associated with the physiological status of host cells. Compared to the phage titer obtained at 3 h (2.77 × 10^10^ pfu/mL), the phage titer was reduced to 0.12 × 10^10^ pfu/mL at 35 h. Generally speaking, in the fermentation process, the host cells grown in the early phase might have the weak cell wall, which was prone to phage infection. As the fermentation entered the late phase, the bacterial cells were in an senile state and DNA started to partly degrade, showing a negative impact on the production of phage (Dong et al. 2013).

In addition to bacterial pellet precipitates, the feasibility of direct incubation, i.e. directly using the fermentation broth for phage infection, was also evaluated. It can be seen from the Fig. 5 that phages with a certain titer were also produced (0.02 × 10^8^-7.94 × 10^10^ pfu/mL). When the fermentation was conducted at 7 h and 9 h, the phage titer reached a high level and then tended to be stable after 9 h. It was obviously not affected by the 1,3-PDO concentration, which has been gradually increasing throughout the fermentation period (Fig. 4). At the end of fermentation (35 h), the glycerol content in the fermentation broth (5.53 g/L) was close to that in the seed medium determined in 3.2, and the other nutrients were also not completely consumed, which was conducive to the infection of phage. Although the phage titer at this time (0.01 × 10^10^ pfu/mL) was lower than that (0.12 × 10^10^ pfu/mL) from bacterial pellet precipitates at the same MOI, this titer could also meet the needs of phage preparation for livestock and poultry with a phage titer of at least 1 × 10^8^ pfu/mL. Nevertheless, the cost of centrifugation was too high for the collection of host cells, and was cumbersome and adverse in the expansion of industrial production scale. Also, membrane filtration used for bacterial sediment had similar disadvantages, which was a continuous cyclic process, accompanied by high pressure and high flow rate (Gill and Hyman 2010). However, the survival rate of host cells in the aging stage at the end of the fermentation would be affected by these factors and further interfered the phage infection. High cost of membrane filtration and treatment of waste water also cannot be ignored. Therefore, direct incubation of fermentation broth for phage infection may be the best choice.

After the comprehensive consideration of 1,3-PDO productivity, phage titer, as well as cost, energy consumption and environmental protection, the direct infection of fed-batch fermentation broth was a proper strategy to co-produce 1,3-PDO and phages.

### Simultaneous separation of fermentation products and phage with two-step SOE

The mixture to be separated was crude phage lysate, which was harvested after direct incubation with phiKpS2 using the fermentation broth at the end of the second fed-batch fermentation (35 h in Fig. 4). The first step of SOE revealed that both 1,3-PDO and phage tend to partition to sodium citrate-enriched bottom phase, whereas the host cells, acetic acid and ethanol were mainly distributed in the top phase. This was consistent with the fact that 1,3-PDO tends to transfer to the salt phase in SOE systems based on hydrophobic solvents (Li et al. 2019b). In addition, previous study have also shown that there is an electrostatic potential difference between the PEG phase and the dextran phase, and the interaction between the positive charge of the PEG top phase and the negative charge of the cell surface causes the cells to be pulled out of the middle phase when salt is used (Cabral 2007). In addition, the partition of bioparticles in a SOE was also related to many other factors, including cell properties (size, net charge, hydrophobicity (Duan et al. 2023), other surface properties (Raja et al. 2011) and the surrounding environmental conditions (salt type and concentration (Duan et al. 2022), organic solvents and concentrations (Duan et al. 2022), pH (Zhang et al. 2020), temperature).

Through the first procedure, the recovery rate of 1,3-PDO and phage in the bottom phase reached to 88.0% and 98.7%, and the removal rate of acetic acid, ethanol and host cells was 93.5%, 91.5%, and 99.4%, respectively (Tables 1 and 2). However, proteins and endotoxins are distributed not only in the middle and top phases but also in the bottom phase in this step. Moreover, other impurities, such as lactic acid, glycerol, formic acid and 2,3-butanediol still coexisted with 1,3-PDO and phages in the bottom phase (Table S3). Thus, the second step of SOE was further carried out.

**Table 1:** Partition behavior of fermentation products during two-step salting-out extraction

Salting-out extraction*		First step	Second step	Total
Recovery of 1,3-propanediol (%)		88.0 ± 1.3	56.6 ± 1.5	49.8 ± 0.6
Partition coefficient (*K*) of 1,3-propanediol		6.2 ± 0.1	2.2 ± 0.04	-
Phase ratio (*Φ*)		1.1	0.6	-
Removal (%)	Lactic acid	0	86.7 ± 1.3	86.7 ± 1.3
	Glycerol	0	73.0 ± 2.0	73.0 ± 2.0
	Formic acid	0	100.0	100.0
	Acetic acid	93.5 ± 1.1	79.9 ± 1.4	98.7 ± 0.2
	2,3-Butanediol	0	0	0
	Ethanol	91.5 ± 2.4	24.2 ± 1.1	93.5 ± 1.9

*The first salting-out extraction system was consisted of 10 wt% sodium citrate/30 wt% ethyl acetate, and n-propanol was then added into the first step bottom phase for the second salting-out extraction

**Table 2:** Partition behavior of phage and impurities during two-step salting-out extraction

Salting-out extraction*		First step	Second step	Total
Recovery of phage (%)		98.7 ± 0.7	97.4 ± 1.1	96.2 ± 0.7
Removal (%)	Proteins	12.1 ± 0.8	94.6 ± 0.6	95.2 ± 0.5
	Cells	99.4 ± 0.5	98.8 ± 0.7	99.9 ± 0.002
	Endotoxin	40.2 ± 0.7	85.5 ± 1.4	91.3 ± 0.8
Volume of phage-rich phase (mL)		5.5	0.01	-
Concentration fold		1.1	550	600

* The two-step salting-out extraction systems were the same as those in Table 1

In the second step SOE, the separation of 1,3-PDO and phage was successfully achieved, in which 56.6% of 1,3-PDO was concentrated to the top phase, and 97.4% of phage was enriched in the middle phase. The partition coefficient and recovery of 1,3-PDO reduced compared with those in the first step SOE (Table 1), which would be improved by multi-stage extraction. According to the theoretical calculation (Türkay and Civelekoğlu 1991), the recovery rate of 1,3-PDO would reach to 99.0% if the extraction were carried out twice. Additionally, n-propanol showed a precipitation effect on phages, which caused the phage to aggregate between the top and bottom phases. Aggregation of phage may be caused by intermolecular hydrophobicity (Zhang et al. 2020) between phages and ionic strength (Asenjo and Andrews 2011; Drab 2018). *E. coli* phage M13 (González-Mora et al. 2017) and T4 (Negrete et al. 2007) also exhibited similar property in ATPSs of PEG400-Na_2_HPO_4_ and PEG600-Na_2_SO_4_. Beneficially, lactic acid, glycerol, formic acid and acetic acid were transferred into the bottom phase (Table S4) with relatively high removal rates of 86.7%, 73.0%, 100.00% and 79.9%, respectively (Table 1). The residual cells, proteins and endotoxin in the first step continued to remain in the bottom phase. Even though 2,3-butanediol still remained together with 1,3-PDO in the top phase, but they could be separated by distillation in the next purification process (Li et al. 2011). Futhermore, 2,3-butanediol is a valuable by-product of the industrial production process of 1,3-PDO (Li et al. 2011).

After two-step SOE, the satisfactory removal rates of lactic acid (86.7%), glycerol (73.0%), formic acid (100.0%), acetic acid (98.7%), ethanol (93.5%), cells (99.9%), proteins (95.2%) were achieved as shown in Tables 1 and 2. It is worth mentioning that endotoxin, a very troubling contaminant with a high toxicity, was removed up to 91.3% (Table 2), which can meet the essential safety in antibacterial phage therapy (Szermer-Olearnik and Boratyński 2015). The recovery rate of phage reached as high as 96.2%. Moreover, the PFs of phage to host cells, proteins and endotoxins were 100,000, 21 and 11 times, respectively, and the total CF of phage in the middle phase were 600 (Table 2). The total recovery of *Klebsiella* phage by SOE in the present study (96.2%) was higher than that in previous study (77%) (Zhang et al. 2020), which used LB medium to prepare phage lysates for SOE rather than fermentation broth. Although the recovery of 1,3-PDO by two-step SOE in the present study (49.8%) was lower than that in previous study (65.6%) (Wu and Wang 2012) using the sodium sulfate/pentanol SOE system, the vast majority of the other fermentation products were removed in the present study, whereas the removal of impurities was not explored in the previous study. In addition, the recovery of 1,3-PDO in the bottom phase would be improved by multi-stage extraction in the second step SOE. Taken as a whole, two-step SOE is very convenient for simultaneous separation of 1,3-PDO and phage from the crude phage lysates. This technique allows efficient removal of impurities and easy access to 1,3-PDO from the top phase, and simultaneously harvest a large number of phage as paste formulation. Furthermore, the separation process is so simplified that some complicated steps can be avoided, which is undoubtedly beneficial to scale up in industrial production of 1,3-PDO and phage. An alternative SOE system could be further developed according to different phages and fermentative products. Our team recently reported that phage phiAB9 was enriched to the middle phase in a one-step SOE system (18% ammonium citrate and 40% ethyl acetate) (Duan et al. 2022), and the recombinant κ-carrageenase and phage T7 could be co-produced by a SOE system composed of 16% ammonium sulfate and 20% ethyl acetate (Chen et al. 2023). These reminded us to explore whether *K. pneumoniae* phage and 1,3-PDO can be simultaneously isolated from the crude phage lysate by one-step SOE. As described in the previous reports (Li et al. 2009a, 2019a; Song et al. 2013; Wu and Wang 2012), the simultaneous isolation of phage and 1,3-PDO can be attempted using a one-step SOE if the fermentation impurities were not considered to be separated at the same time. Otherwise, it is difficult to be achieved by a one-step SOE if the removal of other fermentation impurities was explored as in this study.

## Conclusion

In this work, the reutilization of *K. pneumoniae* after 1,3-PDO fermentation to prepare phages was verified. The MOI of 0.01 and seed medium with 5 g/L glycerol were favorable conditions for phage proliferation. Comparatively, direct incubation of phage in fed-batch fermentation broth was a more economical route for the preparation of phage. Two-step SOE was demonstrated as an efficient means for simultaneous separation and purification of 1,3-PDO and phage. In the first system (sodium citrate/ethyl acetate), 88.0% of 1,3-PDO and 98.7% of phage were gathered in the bottom phase. Then 56.6% of 1,3-PDO and 97.4% of phage were further separately allocated into the top and middle phase in the second SOE system (the bottom phase of the first SOE/n-propanol), respectively. An integral and attractive strategy for co-production and simultaneous separation of 1,3-PDO and phage gives a potential and convenient route for their production on industrial scales.

### Electronic supplementary material

Below is the link to the electronic supplementary material.

**Supplementary Material 1**: **Table S1** *P*-values analyzed by two-way ANOVA for phage titer in different media. **Table S2** *P*-values analyzed by two-way ANOVA for phage titer obtained at different time points of the first fed-batch fermentation. **Table S3** Recovery of fermentation products during the first salting-out extraction. **Table S4** Recovery of fermentation products during the second salting-out extraction. **Fig. S1** The glycerol feeding rate during the second fed-batch fermentation

## Data Availability

The datasets used or analyzed in this study are available from the corresponding author upon reasonable request.

## Abbreviations

1,3-PDO: 1,3-propanediol
CRKP: Carbapenem-resistant < Emphasis Type="Italic”> K. pneumoniae</Emphasis>
SOE: Salting-out extraction
ATPE: Aqueous two-phase extraction
ATPS: Aqueous two-phase system
PEG: Polyethylene glycol
IL: Ionic liquid
BCA: Bicinchoninic acid
MOI: Multiplicity of infection
BSA: Bovine Serum Albumin
