# Supplementary material for: Co-production of 1,3-propanediol and phage phiKpS2 from the glycerol fermentation by Klebsiella pneumoniae
Source: Bioresour Bioprocess. 2024 May 9;11(1):44. doi: 10.1186/s40643-024-00760-w (PMC11082122; doi:10.1186/s40643-024-00760-w)
Supplement: Supplementary file 1 — Supplementary Material 1: Table S1 P-values analyzed by two-way ANOVA for phage titer in different media. Table S2 P-values analyzed by two-way ANOVA for phage titer obtained at different time points of the first fed-batch fermentation. Table S3 Recovery of fermentation products during the first salting-out extraction. Table S4 Recovery of fermentation products during the second salting-out extraction. Fig. S1 The glycerol feeding rate during the second fed-batch fermentation [file 40643_2024_760_MOESM1_ESM.docx]

**Co-production of 1,3-propanediol and phage phiKpS2 from the glycerol fermentation by *Klebsiella pneumoniae***

Suyang Duan^1^, Zhirong Zhang^1^, Xiaoli Wang^1^, Yaqin Sun^1^, Yuesheng Dong^1^, Lina Ren^2^, Lili Geng^2^, Zhilong Xiu^1*^

^1^ School of Bioengineering, Dalian University of Technology, Linggong Road 2, Dalian 116024, P. R. China

^2^ Department of Respiratory, Affiliated Dalian Municipal Central Hospital of Dalian University of Technology, Dalian 116033, Liaoning, P. R. China

*Corresponding author.

Tel./fax: +86 411 8470 6369

E-mail address: [zhlxiu@dlut.edu.cn](mailto:zhlxiu@dlut.edu.cn)

**Key words:** *Klebsiella pneumonia*; 1,3-Propanediol; Bacteriophage; Co-production;

Salting-out extraction

Table S1 *P*-values analyzed by two-way ANOVA for phage titer in different media

| Fermentation time (h) | 5 | 7 | 9 | 11 | 13 | 15 | 18 |
| --- | --- | --- | --- | --- | --- | --- | --- |
| 40g/L vs 20g/L | 0.8284 ns | 0.0030** | 0.7205ns | 0.0442* | 0.3300ns | 0.0024** | <0.0001** |
| 40g/L vs 5 g/L | 0.7790ns | 0.0376* | 0.4881ns | 0.0085** | 0.0001** | <0.0001** | <0.0001** |
| 20g/L vs 5 g/L | 0.9956ns | 0.6038ns | 0.9246ns | 0.7903ns | 0.0093** | 0.1928ns | 0.9004ns |

Note: 40g/L, 20g/L and 5 g/L refer to phage titer in fermentation medium containing 40 g/L glycerol and seed medium with 20 or 5 g/L glycerol, respectively. . *P*-values of less than 0.05 or not were considered to be statistically significant (* or **) or not significant (ns).

Table S2 *P*-values analyzed by two-way ANOVA for phage titer obtained at different time points of the first fed-batch fermentation

| Media | Fermentation medium containing 40 g/L glycerol | Seed medium with 20 g/L glycerol | Seed medium with 5 g/L glycerol |
| --- | --- | --- | --- |
| 5 h vs. 7 h | 0.003** | 0.89ns | 0.29ns |
| 5 h vs. 9 h | 0.75ns | 0.09ns | 0.03* |
| 5 h vs. 11 h | <0.001** | 0.07ns | 0.22ns |
| 5 h vs. 13 h | <0.001** | 0.002** | 0.89ns |
| 5 h vs. 15 h | <0.001** | 0.4ns | >0.99ns |
| 5 h vs. 18 h | <0.001** | >0.99ns | >0.99ns |
| 7 h vs. 9 h | 0.14ns | 0.66ns | 0.94ns |
| 7 h vs. 11 h | >0.99ns | 0.58ns | >0.99ns |
| 7 h vs. 13 h | 0.97ns | 0.06ns | 0.93ns |
| 7 h vs. 15 h | 0.97ns | 0.98ns | 0.5ns |
| 7 h vs. 18 h | 0.54ns | 0.92ns | 0.65ns |
| 9 h vs. 11 h | 0.02* | >0.99ns | 0.97ns |
| 9 h vs. 13 h | 0.01* | 0.82ns | 0.36ns |
| 9 h vs. 15 h | 0.01* | 0.98ns | 0.07ns |
| 9 h vs. 18 h | 0.001** | 0.11ns | 0.12ns |
| 11 h vs. 13 h | >0.99ns | 0.87ns | 0.89ns |
| 11 h vs. 15 h | >0.99ns | 0.97ns | 0.41ns |
| 11 h vs. 18 h | 0.93ns | 0.08ns | 0.56ns |
| 13 h vs. 15 h | >0.99ns | 0.34ns | 0.98ns |
| 13 h vs. 18 h | 0.97ns | 0.003** | >0.99ns |
| 15 h vs. 18 h | 0.97ns | 0.45ns | >0.99ns |

Note: 5 h, 7h, 9 h, 11 h, 13 h, 15 h and 18 g/L refer to phage titer obtained at different time points of the first fed-batch fermentation, respectively. *P*-values of less than 0.05 or not were considered to be statistically significant (* or **) or not significant (ns).

Table S3 Recovery of fermentation products during the first salting-out extraction

| Recovery (%) | 1,3-propanediol | Lactic acid | Glycerol | Formic acid | Acetic acid | 2,3-Butanediol | Ethanol |
| --- | --- | --- | --- | --- | --- | --- | --- |
| Top phase | 12.0 | 0.0 | 0.0 | 0.0 | 93.5 | 0.0 | 91.5 |
| Middle phase | 0.0 | 0.0 | 0.0 | 0.0 | 0.0 | 0.0 | 0.0 |
| Bottom phase | 88.0 | 100.0 | 100.0 | 100.0 | 6.5 | 100.0 | 8.5 |

Table S4 Recovery of fermentation products during the second salting-out extraction

| Recovery (%) | 1,3-propanediol | Lactic acid | Glycerol | Formic acid | Acetic acid | 2,3-Butanediol | Ethanol |
| --- | --- | --- | --- | --- | --- | --- | --- |
| Top phase | 56.6 | 13.3 | 27.0 | 0.0 | 20.1 | 100.0 | 75.8 |
| Middle phase | 0.0 | 0.0 | 0.0 | 0.0 | 0.0 | 0.0 | 0.0 |
| Bottom phase | 43.4 | 86.7 | 73.0 | 100.0 | 79.9 | 0.0 | 24.2 |

**Fig. S1** The glycerol feeding rate during the second fed-batch fermentation.
